# A Study on An EMG Sensor with High Gain and Low Noise for Measuring Human Muscular Movement Patterns for Smart Healthcare

**DOI:** 10.3390/mi9110555

**Published:** 2018-10-29

**Authors:** Sun-Woo Yuk, In-Ho Hwang, Hyeon-Rae Cho, Sang-Geon Park

**Affiliations:** 1 Korea Orthopedics and Rehabilitation Engineering Center, 26 Gyeongin-ro 10beon-gil, Bupyeong-gu, Incheon 21417, Korea; sunwoo@kcomwel.or.kr (S.-W.Y.); ihhwang@kcomwel.or.kr (I.-H.H.); 2 Department of Medical Device Industry; Dongguk University; Goyang-si, Gyeonggi-do 10326, Korea; hrcho721@naver.com; 3 Division of Smart Electrical and Electronic Engineering, Silla University, 140 Baegyang-daero (Blvd.) 700beon-gil (Rd), Sasang-gu, Busan 46958, Korea

**Keywords:** bio-signal, electromyogram, CMRR, phase margin, CMOS technology

## Abstract

The form of the collection of bio-signals is becoming increasingly integrated and smart to meet the demands of the age of smart healthcare and the Fourth Industrial Revolution. In addition, the movement patterns of human muscles are also becoming more complex due to diversification of the human living environment. An analysis of the movement patterns of normal people’s muscles contracting with age and that of patients who are being treated in a hospital, including the disabled, will help improve life patterns, medical treatment patterns, and quality of life. In this study, the researchers developed a smart electromyogram (EMG) sensor which can improve human life patterns through EMG range and pattern recognition, which is beyond the conventional simple EMG measurement level. The developed sensor has a high gain of 10,000 times or more, noise of 500 uVrms or less, and common mode rejection ratio (CMRR) of 100 dB or more for EMG level and pattern recognition. The pattern recognition time of the sensor is 30 s. All the circuits developed in this study have a phase margin of 75 degrees or more for stability. Standard 0.25 μm complementary metal oxide semiconductor (CMOS) technology was used for the integrated circuit design. The system error rate was confirmed to be 1% or less through a clinical trial conducted on five males in their 40s and three females in their 30s for the past two years. The muscle activities of all subjects of the clinical trial were improved by about 21% by changing their life patterns based on EMG pattern recognition.

## 1. Introduction

In recent years, people’s interest in health has increased, and the desire to receive a personalized diagnosis according to each individual’s health condition is increasing. In addition, such self-diagnosis is becoming more intelligent and smart, affecting people’s life patterns. In this healthcare industry, “customized precision medical care” is not a mere fashion but a core keyword of the future Fourth Industrial Revolution.

As a result, even companies that have nothing to do with recent medical care are strategically focusing investments in “bio” and “healthcare.” In particular, the biosensor is capable of “on-site diagnosis” that enables confirmation of the presence or absence of diseases by simple operation anytime and anywhere with only a small micro- or nanoscale sensor, and the field of combination with smart wearable devices is particularly conspicuous. The global self-diagnosis market size exceeded 25 trillion Korean won in 2017. The biosensors currently in the world market include a pressure sensor that is used as a blood pressure monitor, a silicon microphone that is used for high sensitivity hearing aids, a pacemaker accelerometer, and a microfluidic sensor used in capsule endoscopes. Accordingly, this study introduces a micro-sized integrated electromyography sensor as a biosensor.

The existing electromyography sensor measures the current signal of the human body outputted according to the contraction and relaxation state of the muscles through electrodes on the skin surface of the human body [1]. In order to measure various signals, a plurality of sensors are necessary, and in order to obtain a lot of information, the design size has only to be increased. Also, common component noise induced to the physical skin can be included, and the electromyogram signal itself is also considerably weaker than noise, so a high output, low noise design is required [2]. The existing design was meant to be used only in hospitals for reasons that the measurement form should be detailed and precise, but in order to combine it with smart diagnosis and wearable devices, it is necessary to design it with ultra-compactness by using integrated circuit technology [3]. Therefore, in this research, we designed an smart electromyogram (EMG) sensor that is highly resistant to noise while having a high amplification factor by using an ultra-small integrated circuit and complementary metal oxide semiconductor (CMOS) technology.

## 2. System Overview

### 2.1. Block Diagram of the EMG Sensor

**Figure A1:**
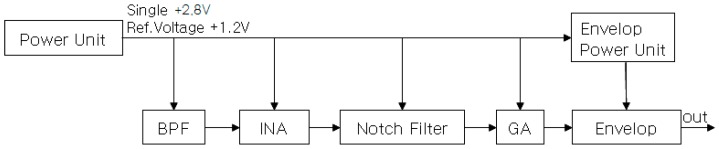
Block diagram of the smart electromyogram (EMG) sensor.

Figure A1 shows the block diagram of the sensor used in this study [4,5]. There is a power unit that applies power to each block, a band pass filter (BPF) to remove input terminal noise, an instrumentation amplifier (INA) for high power, a notch filter for 60 Hz noise removal, a gain amplifier (GA) for power factor adjustment, and a low-pass filter envelope. The operating range is 0 to 2.8 V, the operating frequency is 90 to 450 Hz, and the total gain is 100,000 times, excluding the envelope stage of the nonlinear amplification stage.

### 2.2. ASIC of Core-Amplifier

Figure A1 shows the block diagram of the sensor used in this study [4,5]. There is a power unit that applies power to each block, a band pass filter (BPF) to remove input terminal noise, an instrumentation amplifier (INA) for high power, a notch filter for 60 Hz noise removal, a gain amplifier (GA) for power factor adjustment, and a low-pass filter envelope. The operating range is 0 to 2.8 V, the operating frequency is 90 to 450 Hz, and the total gain is 100,000 times, excluding the envelope stage of the nonlinear amplification stage [6,7].

**Figure A2:**
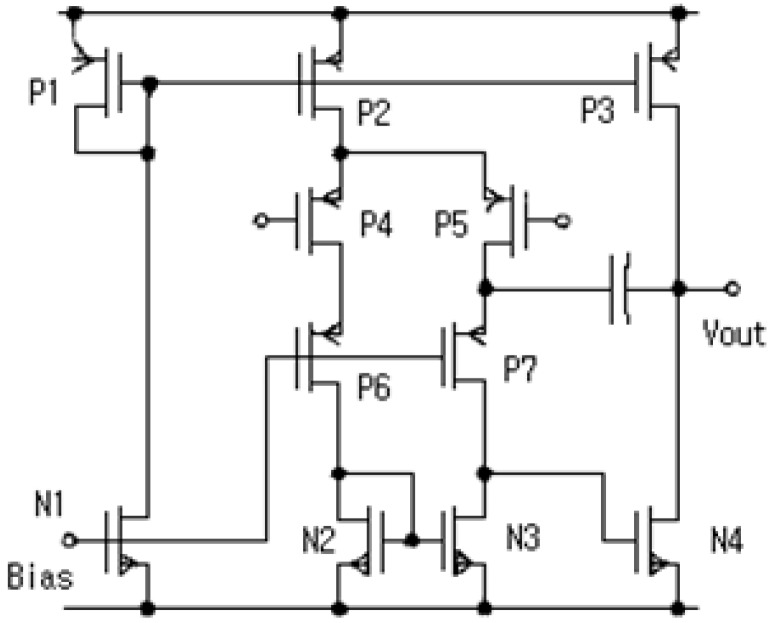
Schematic of core-amplifier.

The charge sensitive amplifier has been designed with a traditional scheme, as shown in Figure A2. It consists of a low noise integrator and low impedance output buffer to drive the EMG signals. Both the preamplifier and output buffer are based on a single- ended folded cascade structure. This solution offers better performance in terms of stability and gain compared with a standard cascade. The p-channel metal-oxide-semiconductor (PMOS) transistor of the input stage will be optimized for 1/f noise. However, we expected the noise to be dominated by the series thermal noise of the input transistor, so we selected an n-channel metal-oxide semiconductor (NMOS) device which provides higher transconductance than the complementary PMOS at the same drain current [8,9].

The width and length parameters of each metal-oxide-semiconductor (MOS) component used in the amplifier were iterated to optimize amplification factor and power consumption. The integrated circuits (IC) manufacturing process has a threshold voltage of about 0.6 V for PMOS and NMOS components. Some performance characteristics for the IC are summarized in Table 1.

The noise analysis form was important in the design of the width of P4 and P5, the length of N2 and N3, and the ratio of the length of N2 and N3 to the length of P4 and P5. The final design parameters were chosen to maximize the amplifier’s performance in switched capacitor circuits. The input devices P4 and P5 are cascaded with P6 and PI, which have no significant effect on noise performance. These cascade devices improve the gain of the input stage and allow for an unusual connection of the compensation capacitor at the common source-drain node. The node has a gain of less than unity from the amplifier inputs, which ensures that the right-half plane zero caused by feed forward through the compensation capacitor will not cause oscillation [10]. This method of compensation is less sensitive to loading on the second gain stage than other methods. This permits the output to be taken directly from the second stage without the addition of an output stage [11]. It is not intended for this amplifier to drive resistive loads or large external capacitances. The dimensions of the opamp are shown in Table 2.

### 2.3. Noise Analysis of Core-Amplifier

The limit of resolution of a sensor is often determined by noise. We analyzed the noise source data to extract the key parameters for each of the three noise sources [12].

#### 2.3.1. 1/*f* Noise

At low frequencies, 1/*f* noise can be dominant. However, at high frequencies, 1/*f* noise drops below thermal noise. We can do two things to minimize 1/*f* noise. One is to reduce the device area, W/L, and the other is to use a buried channel device to separate the channel from the interface. While in the standard CMOS process we cannot do much about the second option, we can reduce W/L. However, the gain of the amplifier is dependent on W/L. Therefore, reducing L is the best choice. The 1/*f* noise has a noise spectral density as defined in Ref [7], $e_{1/f}=\sqrt{k_{f}/({C_{\mathrm{ox}}}^{2}WLf^{\alpha })}$, where, $k_{f}$ is the flicker noise coefficient and α is close to 1 [12].

#### 2.3.2. Serial Thermal Noise

Serial thermal noise is due to the random thermal motion of the free carriers in the channel of a MOSFET. It can be expressed as a series voltage source given by Equation $e_{\mathrm{thermal}}=\sqrt{4kTR_{n}B_{n}}≅\sqrt{4kTR_{s}{C_{i}}^{2}/\tau }$, where, $R_{S}$ is the equivalent series thermal noise resistance, $C_{i}$ is the total capacitance at the input, and τ is the amplification time. There, the total input capacitance of the amplifier adds its differential input capacitance and common mode input capacitance, making $C_{i}=C_{d}+C_{id}+C_{icm}≅C_{d}$. Bn is noise equivalent bandwidth. This is defined as the voltage-gain-squared point at which the two shaded areas are equal. So, we must reduce the noise gain bandwidth to reduce thermal noise [10].

#### 2.3.3. Parallel Noise

Parallel noise is expressed by equation $e_{p}=\sqrt{2kT\tau /R_{p}}$. When the feedback network is in a higher resistance condition, the parallel noise vanishes.

**Figure A3:**
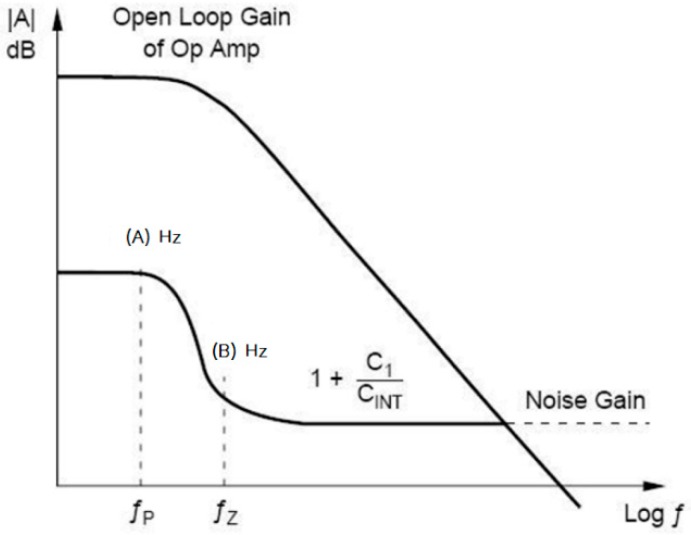
Noise gain response of core-Amplifier.

The noise gain response for the amplifier during integration operating mode is shown in Figure A3.

The low frequency pole of the noise gain is equal to:

(1) $$f_{p}=\frac{1}{2\pi R_{f}C_{\mathrm{int}}}≅\frac{1}{2\pi (R_{\mathrm{open}}+R_{f})C_{f}}$$

This pole is usually found at very low frequencies.

The zero frequency of the noise gain plot is equal to:

(2) $$f_{z}=\frac{1}{2\pi (R_{\mathrm{sh}}\|R_{f})(C_{i}+C_{\mathrm{int}})}≅\frac{1}{2\pi (R_{\mathrm{sh}}\|(R_{\mathrm{open}}+R_{f})(C_{d}+C_{f})}$$

(3) $$\mathrm{High}\;\mathrm{frequency}\;\mathrm{noise}\;\mathrm{gain}=(1+\frac{C_{i}}{C_{f}})$$

#### 2.3.4. Design of EMG Sensor

**Figure A4:**
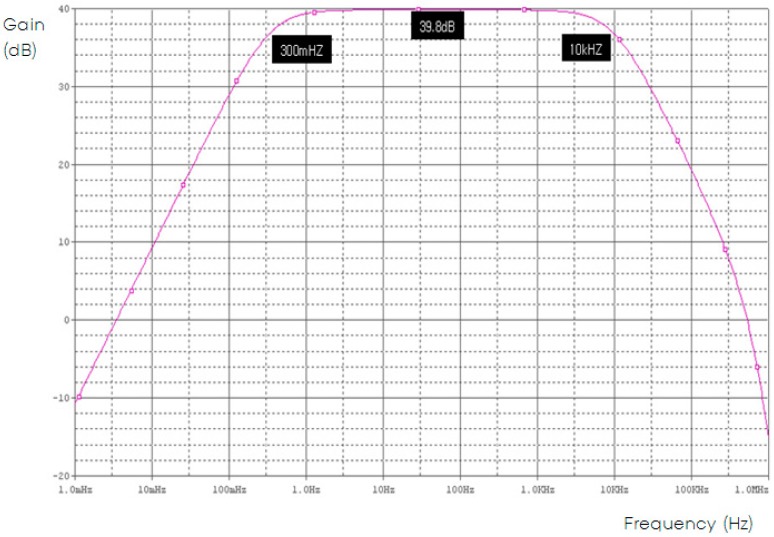
Output voltage gain and frequency band of the instrumentation amplifier (INA) stage of the core-amplifier.

The first part between human body skin and the circuit was designed with an Instrumentation Amplifier. The signal detected at the two input electrodes of the electrode is used as the input signal of the differential amplifier, and the reference electrode is connected to the ground of the differential amplifier to constitute a common ground of the sensor power and the detected electric potential signal. The frequency and gain ratio of the instrumentation amplifier stage are shown in Figure A4 The total gain is 100 times (40 dB) compared with the input signal, and has a signal bandwidth of about 300 mHz to 10 kHz. In addition, it has been designed to have a high common-mode rejection ratio (CMRR) of over 100 dB, and at the same time, to have an excellent signal-to-noise ratio (SNR) [13]. The notch filter stage uses a one-order sallen-key notch filter. For accurate frequency rejection, passive components (resistors and capacitors) with a tolerance of 1% should be used. Gain is 3/2 times (3.5 dB) compared with the output signal of INA, and frequency band rejection occurs at 60 Hz. In addition, by using a two-stage gain amplifier and gain adjuster as a variable resistor, it is possible to adjust the signal size according to the EMG signal, and the offset voltage can be easily removed by using a direct current (DC) blocking capacitor. Since the strong local electric potential signal appears in the frequency band between 50 Hz and 120 Hz, it is designed to output the maximum output ratio in the frequency band [14,15].

## 3. Result and Discussion

### 3.1. Myoelectric Signal Analysis Results

**Figure A5:**
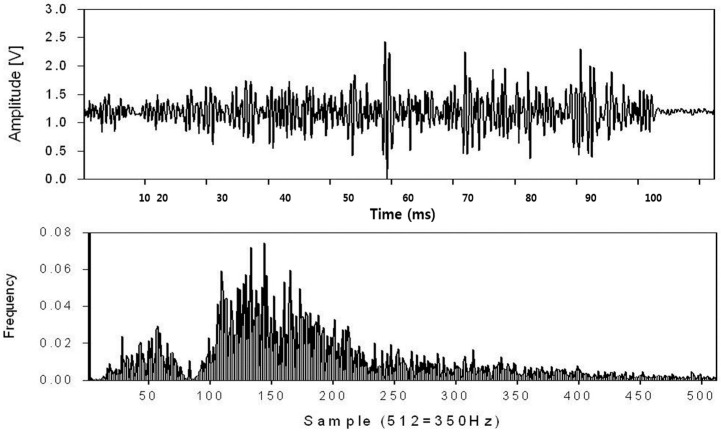
Measured raw EMG signal and fast Fourier transform (FFT) signal.

**Figure A6:**
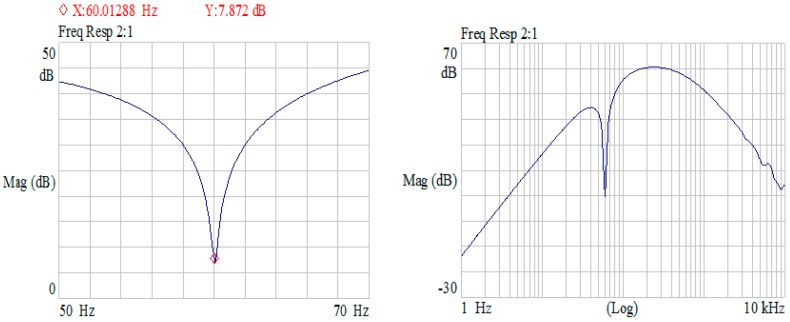
Notch filter stage of the EMG sensor and the measured frequency of the final output stage.

Figure A5 shows the raw EMG signal and frequency band collected by the EMG sensor. Figure A6 shows the 60 Hz frequency band removed through the sallen-key notch filet and the final frequency band output from the sensor. In both figures, the output is very high, i.e., in the 80 Hz to 150 Hz frequency range, which is the main band of the EMG signal. Also, by having an output value of about 7.8 dB in the 60 Hz band, it can be seen that the 60 Hz signal is removed by about 1/5 of the output signal [16,17]. Also, it can be seen that the noise components in the low frequency band and the high frequency band are completely removed through the bandpass filter [18,19].

### 3.2. Noise Analysis Results

**Figure A7:**
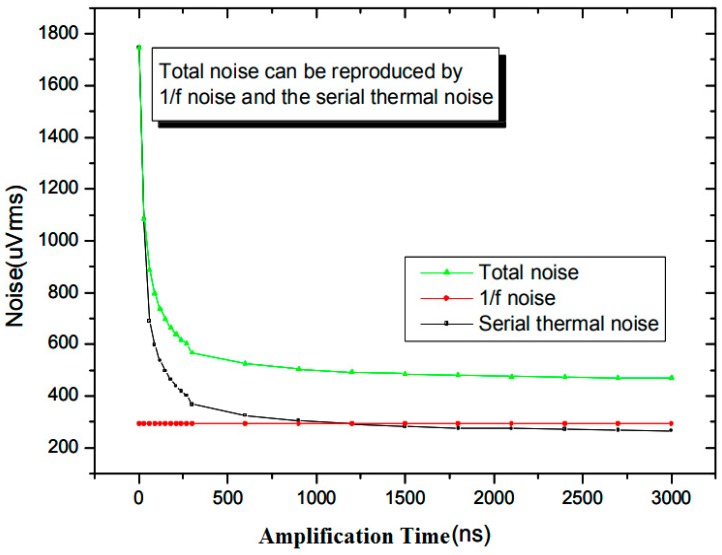
Total noise versus amplification time.

Figure A7 shows the total noise characteristics across the circuit due to 1/*f* noise and thermal noise. The current sensor is attached to human skin and can be affected by sweat, moisture, and temperature. Therefore, it should be checked whether the input waveform of the sensor and the temperature change of the whole sensor are influenced by the output waveform. If the signal is saturated, it can be seen that phase noise has occurred for a fine input signal, which is usually due to the thermal noise of the transistor of the active device [20,21]. As a result, 1/*f* noise increases at low frequencies. In other words, oscillation occurs, the phase shifts slightly in the time axis waveform of the signal, and the waveform looks distorted, eventually leading to saturation. In addition, since the resistance value across the circuit is affected by the capacitance of the skin of the human body, thermal noise is generated, which is gradually lowered when the circuit is stabilized. Also, in this circuit, the parallel noise is very large because the feedback network has gone through several stages due to high amplification. Therefore, only 1/*f* noise and serial thermal noise remain.

**Figure A8:**
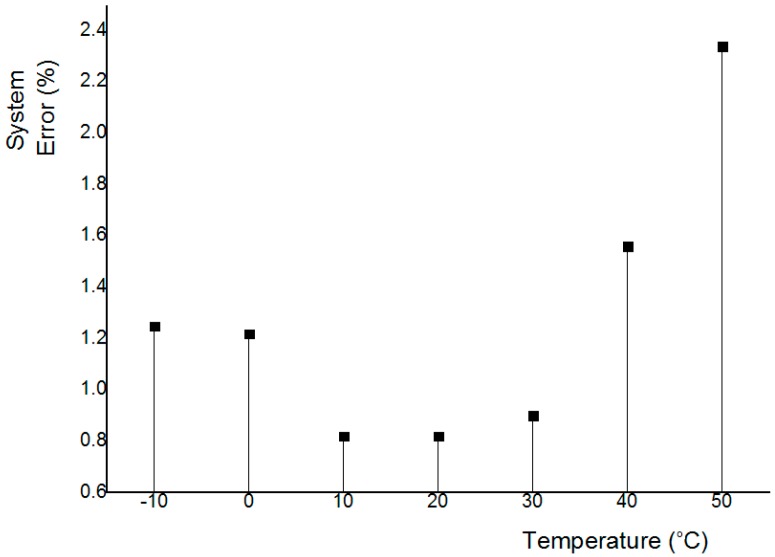
System error rate for temperature rise.

As shown in Figure A7, the designed circuit generates a total of 500 uVrms after the stabilization time, and the result is less than 1 mVrms at the human body signal collection sensor. Figure A8 shows the system error rate with temperature, and the system error rate is below 1% at room temperature. Here, system error rate means that as the temperature rises, a signal that is difficult to obtain by the EMG signal comes out. Typically, analog asic is 1/*f* noise, so the noise rises as the temperature rises. This also shows that this system is relatively strong against 1/*f* noise.

Gain and phase margin are a measure of the stability of the semiconductor sensor. Usually, phase margins are more useful than gain margins in measuring system stability. In general, a semiconductor sensor has a target phase margin of 60 degrees and must maintain at least 45°. Table 3 shows the error rate of the EMG sensor for the phase margin. Here, the phase of signal phase is set based on 0.2 ms. If the phase changes by more than 0.2 ms, the stability of the electromyogram sensor will decrease [22]. Table 3 shows the system error rate for the phase margin and phase signals. The sensor has a phase margin of 65 degrees, a signal phase of more than 0.2 ms, a noise level of about 500 uVrms, and a system error rate of more than 1%. Therefore, this sensor must use a phase margin within 65 degrees. If this sensor is used more than this, it may receive the distorted signal of the system itself in addition to the EMG signal [23,24].

### 3.3. Verification of Results of Research

Robust statistics were used to verify the reliability of the study results. In particular, when the result value fluctuates like a bio-signal, it is necessary to verify the resultant value and the analysis data by using such a statistical technique. First, the robust verification of voltage gain, noise measurement, and system error rate is shown in Table 4. As a result of 10 test repetitions, the Z-scores were 0.83, 0.96, and 0.98, respectively, showing more than 95% confidence in the test results. [& = Test results are very consistent or satisfactory = Reliability > 95%]. Reliability verification of the system error rate was performed at room temperature. According to Table 3 and Table 4, we can see that the system error rate and noise are almost the same as those of the data on the phase margin and the robust statistics.

### 3.4. Clinical Trial Results

Clinical trials were conducted on five male patients in their 40s and three female patients in their 30 s. The clinical trial procedure is shown in Table 5.

**Figure A9:**
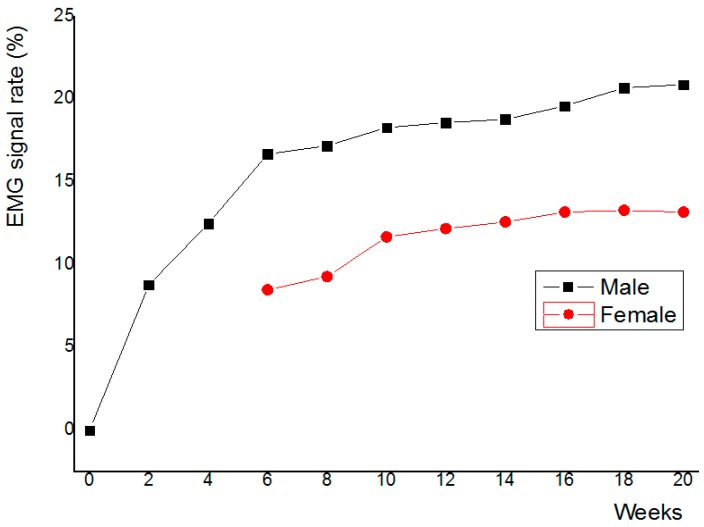
Improvement of muscle movement activity with EMG sensor.

**Figure A10:**
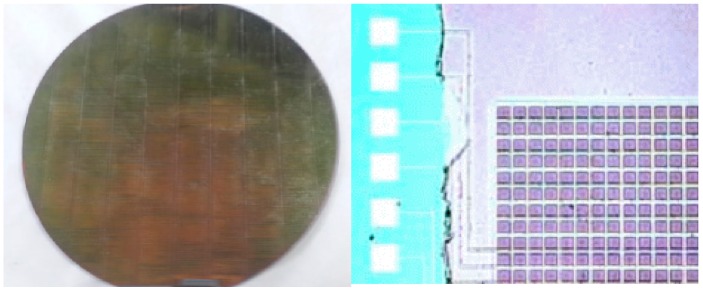
Scanning electron microscopy (SEM) photo of semiconductor wafer and some patterns.

A preliminary study was performed on the patient while testing the signal and error rate of the sensor and the Institutional Review Board (IRB) approval number is RERI-IRB-180921. The selection criteria for the study subjects were upper limb amputees, and the remaining muscle signals were selected as measurable. We also chose people without such a condition through questions and answers. For about 20 weeks, from February 2017 to June 2017 for male patients and from April to 2017 for female patients, the sensor was attached for about 16 weeks until June, and it was confirmed that it operated normally. However, the system error rate increased with body temperature rise and ambient temperature rise, and further research has been carried out to reduce the error rate of the temperature rise. Figure A8 shows the system error rate with temperature, and the system error rate is below 1% at room temperature. Figure A9 shows the improvement of muscle movement and activity until the end of the test by recommending the use of unused muscles by detecting the signals of muscles every two weeks. Figure A10 shows a SEM photo of the designed semiconductor wafer and some patterns [27,28].

## 4. Conclusions

The theme of this study is the integrated circuit design of a high-amplification, low-noise EMG sensor for self-diagnosis and efficient use of muscles in the era of smart healthcare.

The research result was to make an operational amplifier (op amp) sensor by using 0.25 micron CMOS technology, high gain and low noise, and artificial intelligence. The most intensive part of the op amp design was for the acquisition of signals at around 100 Hz, which is the main frequency of the full-revolution signal, with a very small number μA. In particular, due to the nature of the biometrics, it was vulnerable to low frequency bands and 60 Hz noise, so we were concerned with the design of a notch filter for the elimination of 60 Hz and reduction of 1/*f* noise for total noise reduction. Also, the 95% confidence level was finally tested. However, the increase in system error accompanying the temperature rise and the title of the clinical trial considers another idea of research. It causes a thermal shock, raises the temperature, and has a serious effect on it. Therefore, we need to do further research to improve it.

We hope that the results of this study will improve the quality of life of humans by offering alternative life patterns through EMG pattern recognition, and improve muscle activity for patients who are recovering from accidents.

## Figures and Tables

**Table 1:** Performance characteristics of core-amplifier.

Parameter	Existing Product (Ottobock)	Value (Manufactured EMG Sensor)
**Gain**	By feedback resistance	10 mV/fC
**Noise**	477 uVrms	500 uVrms
**Output Swing**	5 V	2.8 V
**GBW (gain bandwidth)**	10 kHz	200 MHz
**Input Range**	10 fC	4 fC
**Linearity**	0.3% S.d	0.3% S.d
**Power Consumption**	About 2 mW	2 mW
**CMOS Technology**	N/A	0.25 μm

**Table 2:** Dimensions of the Core-Amplifier.

Device	Z/L	Device	Z/L	Device	Z/L
**P1**	60/20	**P1**	50/10	**N2**	120/75
**P1**	350/20	**P1**	120/10	**N3**	120/75
**P1**	300/20	**P1**	120/10	**N4**	60/20
**P1**	50/10	**N1**	10/10	-	-

**Table 3:** System error rate for phase margin and phase signal.

Phase Margin	Phase of Signal	Noise	% Error Rate
45°	0.134 ms	145 uVrms	0.23%
50°	0.145 ms	195 uVrms	0.43%
55°	0.168 ms	286 uVrms	0.75%
60°	0.198 ms	369 uVrms	0.98%
65°	0.226 ms	469 uVrms	1.08%

**Table 4:** Reliability verification through robust statistics.

Parameter	Gain	Noise	System Error
Number	10	10	10
Avg	9.8 mV/fC	487 uVrms	1.11%
Median	9.9 mV/fC	502 uVrms	1.03%
Z-score	0.83	0.96	0.98

**Table 5:** Clinical Trial Procedures.

1. The EMG signal is measured for the upper limb cutter.
2. Research participants should change to the same lab uniform. To measure the EMG of the upper extremity muscles, use the EMG sensor where the upper extremity moves most strongly. In addition, the other hand is grounded to obtain accurate experimental measurement data. Do not collect signals whose signal levels are shaken or whose thresholds are not exceeded [25,26].
3. All experimental data use only data values that meet 95% confidence with robust statistical processing techniques.
4. Experimental method A. EMG measurement for 5 min in rest (sitting comfortably) B. Perform the same type of measurement every week at the right time C. Assessment of whether all data satisfy 95% confidence level in robust statistics D.Measure muscle movement (It is measured by the EMG signal level because it is impossible to measure with the naked eye.) E. Record the temperature rise of the system at all measurements. F. Record system error rate as temperature increases G. Record until after the system has an idle period until the error is less than 1%. H. The same type of measurement should be performed at intervals of 2 weeks for 20 weeks.
